# Evolutionary Origin of Ocular Melanoma: Associations With rs12913832 G Allele Frequency and Latitude

**DOI:** 10.1002/cam4.72038

**Published:** 2026-06-11

**Authors:** Hans Witzenhausen, Hildur Helgadottir, Veronica Höiom, Roger Olofsson Bagge, Gustav Stålhammar

**Affiliations:** ^1^ St. Erik Ophthalmic Pathology Laboratory St. Erik Eye Hospital Stockholm Sweden; ^2^ Department of Oncology and Pathology Karolinska Institutet Stockholm Sweden; ^3^ Theme Cancer Karolinska University Hospital Stockholm Sweden; ^4^ Sahlgrenska Center for Cancer Research, Department of Surgery, Institute of Clinical Sciences, Sahlgrenska Academy University of Gothenburg Gothenburg Sweden; ^5^ Department of Surgery Sahlgrenska University Hospital Gothenburg Sweden; ^6^ Wallenberg Centre for Molecular and Translational Medicine University of Gothenburg Gothenburg Sweden; ^7^ Department of Clinical Neuroscience, Division of Eye and Vision Karolinska Institutet Stockholm Sweden; ^8^ Ocular Oncology Service St. Erik Eye Hospital Stockholm Sweden

**Keywords:** blue eyes, conjunctival melanoma, genetic risk, incidence, latitude, uveal melanoma

## Abstract

**Background:**

Ocular melanoma (OM) incidence varies worldwide, but the relative impact of geographic latitude versus pigmentation genetics remains debated. We investigated whether the country‐level frequency of the rs12913832 derived (G) blue‐eye allele (G allele frequency), or latitude better explains international differences in OM incidence.

**Methods:**

We performed an observational ecological study of 29 countries using age‐standardized OM incidence data from the International Agency for Research on Cancer (IARC), G allele frequencies from publicly available databases, and population‐weighted centroids derived from nighttime lights data. We conducted correlation, linear regression, and mediation analyses to assess the associations among latitude, G allele frequency, and OM incidence.

**Results:**

In univariate analyses, both latitude and the G allele frequency were positively associated with OM incidence. In multivariate regression, latitude was no longer significant, whereas the G allele frequency explained substantially more variance and uniquely accounted for 61 times more variance (partial *r* ^2^) than latitude. Mediation analysis indicated that about 92% of latitude's effect on incidence was mediated through G allele frequency.

**Conclusion:**

At the population level, the G allele frequency is a markedly stronger predictor of OM incidence than geographic latitude. As an ecological analysis, this study identifies a population‐level association rather than individual‐level genetic risk and suggests that pigmentation genetics underlie much of the international variation in OM incidence.

## Graphical abstract

The rs12913832 derived (G) allele frequency (blue‐eye allele) was a markedly stronger predictor of ocular melanoma incidence than geographic latitude, with approximately 92% of the latitude effect mediated through allele frequency. These findings indicate that pigmentation‐related genetic variation contributes substantially to global differences in ocular melanoma risk.

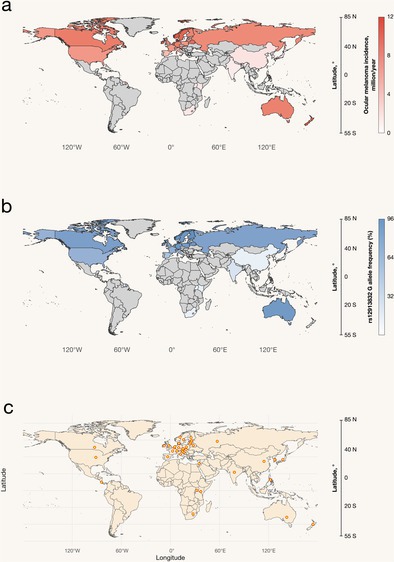

## 1 Introduction

Ocular melanoma (OM) can be divided into uveal and non‐uveal melanoma. Uveal melanoma (UM) comprises the vast majority of OM (approximately 85%) and includes choroidal, ciliary body, and iris melanoma [1]. Several pigmentation‐related risk factors are associated with UM, including light iris color, fair skin color, limited ability to tan, atypical cutaneous nevi, common cutaneous nevi, cutaneous freckles, and iris nevi [2, 3].

The single nucleotide polymorphism (SNP) rs12913832 is widely recognized as the primary genetic determinant of blue eye color in humans [4]. It is located within intron 86 of the *HERC2* gene on chromosome 15, approximately 21 kilobases upstream of the *OCA2* promoter [4]. This variant acts as a regulatory element modulating *OCA2* expression, which in turn influences melanin production in the iris [5]. Throughout this manuscript, rs12913832 genotypes are denoted AA, AG, and GG, where G represents the derived allele. The ancestral allele (A) promotes normal *OCA2* activity, resulting in higher melanin levels and brown eye color [4, 6]. In contrast, the derived allele (G) disrupts a regulatory enhancer, reducing *OCA2* expression and melanin production, and thereby producing blue irises, most strongly in homozygous carriers (GG) [4, 6]. This recessive effect is highly specific; nearly all blue‐eyed individuals carry the derived allele on a shared haplotype, confirming that rs12913832 is the causal variant for blue eye color [4]. Beyond its role in eye pigmentation, the rs12913832 G allele has been directly associated with uveal melanoma risk in candidate‐gene association studies and in genome‐wide association studies, where it shows a particularly strong association with monosomy 3 tumors [7, 8].

Available Neandertal genomes indicate that the derived allele at rs12913832 is absent in Neandertals and other archaic hominins, supporting the notion that this mutation arose within modern *Homo sapiens* . Multiple analyses, including those based on high‐coverage Neandertal genome sequences (Altai and Vindija), consistently fail to detect this variant, indicating that the derived (G) allele at rs12913832 emerged after the divergence of modern humans and Neandertals [9, 10, 11].

Genetic analyses suggest that the derived allele arose approximately 6000–10,000 years ago during the Neolithic period [4, 12]. Haplotype studies show that nearly all blue‐eyed individuals share a common ancestor harboring this mutation, implying a single founder event in modern humans [4]. The mutation appears to trace back to the Black Sea region, at the borderlands of Europe and Asia. It likely spread throughout Europe with Neolithic migrations, potentially driven by founder effects and selective pressures such as sexual selection or adaptation to northern latitudes [4, 12].

The incidence of UM varies considerably by geographic region and has been reported to increase with distance from the equator, particularly in populations of predominantly European ancestry and in datasets with high case verification [13]. In Africa, uveal melanoma is exceedingly rare, and in Asia, its incidence per million person‐years is < 1, compared with > 10 in Northern Europe [13, 14, 15]. Although ultraviolet (UV) light has been proposed as a risk factor, the evidence remains inconsistent [16, 17, 18]. Recent studies do not identify a UV‐radiation signature in choroidal or ciliary body melanoma, in contrast with iris and conjunctival melanoma [19, 20]. One study reported a higher incidence of UM in the northern versus southern United States, even when restricted to non‐Hispanic whites [21]. However, the prevalence of blue eyes may vary by latitude among non‐Hispanic whites. Consequently, some point to the geographical distribution of the blue‐eye allele as a possible underlying driver [13].

In this study, we collected data on country‐level rs12913832 derived (G) allele frequency (G allele frequency), OM incidence, and latitude across multiple countries to evaluate whether G allele frequency or latitude better explains the observed differences in incidence.

## 2 Methods

### 2.1 Inclusion Criteria

- Countries reporting age‐standardized OM incidence data from reputable sources such as the International Agency for Research on Cancer (IARC).
- Availability of G allele frequency data from systematically curated databases for the corresponding country populations.

### 2.2 Exclusion Criteria

- Countries with incomplete or missing data on OM incidence or G allele frequency.
- Data from populations with overlapping or duplicate entries and cases with unknown age at onset of OM.

### 2.3 Incidence Rates

Age‐standardized incidence rates for 36 countries were obtained from a previous publication on the worldwide incidence of OM, which in turn had collected data from IARC [13, 22]. All cases were microscopically verified, and unspecified malignant neoplasms were reallocated to OM as previously described [23]. To remain consistent with earlier reports from Africa and South America—both of which had a 100% microscopic verification rate—cases from these regions were not reallocated. OM cases of unknown age of onset (*n* = 36) were excluded. Duplicate data and overlapping populations were excluded, as described previously [13].

### 2.4 Allele Frequencies

Data on the G allele frequency were obtained from the Allele FREquency Database (ALFRED) maintained by Yale University [24, 25]. This resource compiles published allele frequency data that have been systematically curated for over 20 years. Of the 36 countries with incidence data, allele frequencies were available for 26. For populations with multiple entries (e.g., Danes, Finns, Russians, Italians, British, Spaniards, European Americans), we calculated the arithmetic mean of the reported allele frequencies. For instance, two Danish values of 87% and 84% yielded an average of 86%, while 13 Italian entries averaged 50%. For China, we used the G allele frequency reported for the Han population, which accounts for 91% of the country's total population [26]. In many countries, the allele frequencies reported for specific ethnic groups do not necessarily reflect the overall G allele frequency for the country. Therefore, we estimated weighted averages by combining the G allele frequency data from these groups with their respective proportions in the total population. For example, in Israel, we averaged the allele frequencies reported for Jews, who comprise 74% of the population (with 47% being Ashkenazi [G allele frequency 57%], 30% Sephardic [G allele frequency 54%–56%], and 23% Mizrah [G allele frequency 16%]), while 21% of the population are Arab (approximate G allele frequency 20%) [26, 27]. Based on these data, the overall weighted average G allele frequency for 95% of the Israeli population is 42%. For Lithuania, ALFRED provided no data; instead, we relied on a publication by Urnikyte et al. [28], which reported 92% G allele frequency—84% being homozygous—among families resident in Lithuania for at least three generations. Considering the ethnic distribution, we calculated a weighted average frequency of 82%. For Canada, separate frequencies of 63% in Quebec, 77% in the Atlantic provinces, 77% in Alberta, and 76% in both British Columbia and Ontario resulted in a weighted average of 73% [29]. For Belarus, a frequency of 82% has been reported [30]. Altogether, G allele frequencies were thus available for 29 countries. A full list of weighted averages, alongside each country's ethnic breakdown, appears in Table S1.

### 2.5 Population Centroids

We extracted country‐level population‐weighted centroids for the 29 countries with available data on G allele frequency from the 2019 project by Hall and colleagues [31]. These centroids represent the average geographic position of a country's population, accounting for internal variation in population density. Unlike geometric centroids, which mark the geographic center of a country's landmass, population centroids aim to identify the point around which the population is balanced—analogous to a center of mass. Specifically, the latitude and longitude coordinates represent the location such that half of the population lives north and half south of it, and half east and half west, based on nighttime lights data as a proxy for population distribution.

**FIGURE 1:**
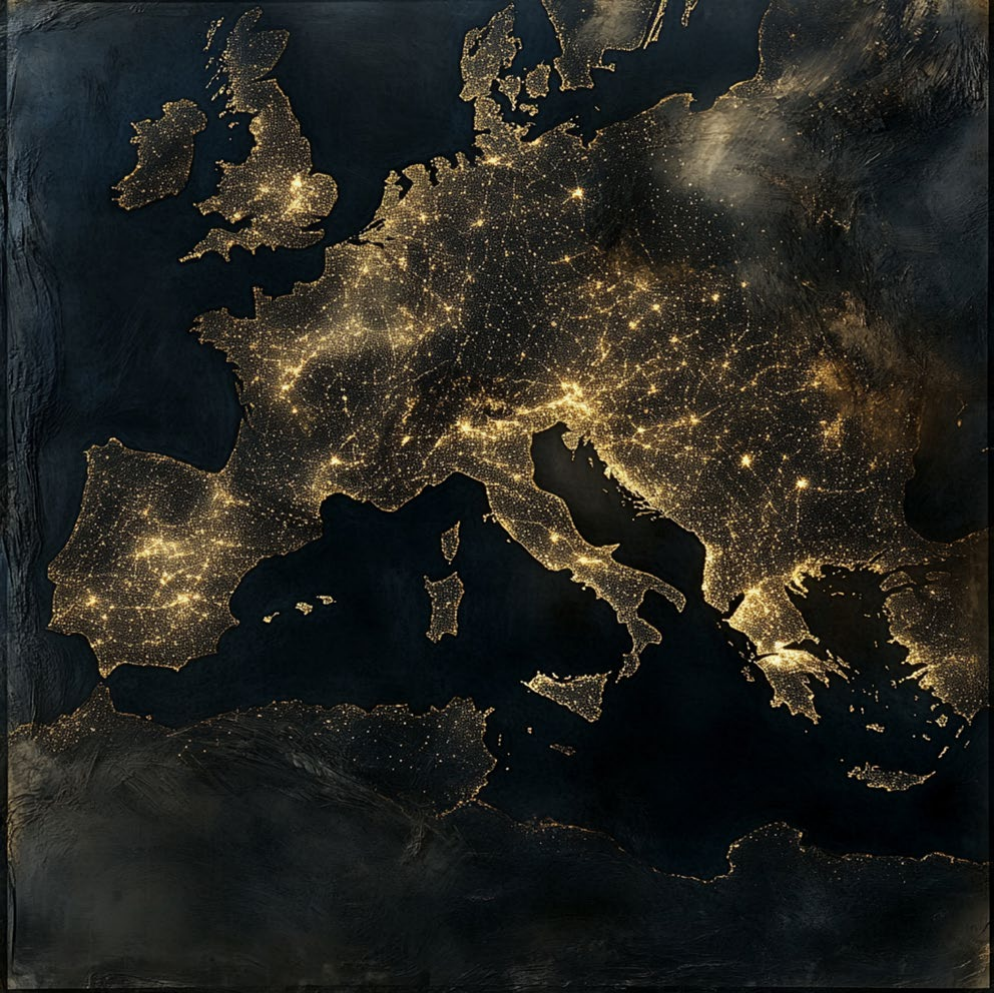
Conceptual representation of the process for generating country‐level population centroids. For 29 countries with available data on the rs12913832 G allele frequency, calibrated nighttime lights imagery was used to estimate population centroids. Using the Aboufadel and Austin method, light intensities were weighted to compute spherical means of georeferenced coordinates, yielding centroids that represent the population's balance point (i.e., half the population lies north and south and east and west of the computed center), rather than the geometric center of the landmass.

We used the dataset *Population centroids of the world 1992–2013*, compiled by Hall et al. and available at Figshare (https://doi.org/10.6084/m9.figshare.9939494). This dataset provides annual centroid estimates from 1992 to 2013 for each country using the method developed by Aboufadel and Austin, which calculates spherical weighted means of georeferenced light intensity data derived from Defense Meteorological Satellite Program (DMSP) imagery. Estimates for the year 2013 were used. Nighttime light intensity was used as a proxy for human settlement and population density, and the centroids were calculated by weighting spatial coordinates according to light intensity within each country (Figure 1). Coordinates were then converted to latitude and longitude using great‐circle geometry to reflect the curvature of the Earth.

### 2.6 Ethical Considerations

This study was conducted in accordance with all applicable federal laws and institutional regulations. According to the Swedish Ethical Review Act, ethical approval is required if research involves physical intervention, on living and deceased persons alike; is carried out with a method that aims to affect the research participant physically or mentally, or involves an obvious risk of harm to them in body or mind; are performed on biological material from a living or deceased human being and can be traced back to that person; or involves processing of sensitive personal data or of personal data relating to criminal offenses [32]. None of these criteria apply to the current study, which exclusively uses publicly available, aggregated country‐ and population‐level data on ocular melanoma incidence, allele frequencies, and latitude. Consequently, ethical approval was not required. The Strengthening the Reporting of Observational Studies in Epidemiology (STROBE) checklist was adhered to.

### 2.7 Statistical Analyses

All *p* values were two‐sided, and *p* < 0.05 was considered significant. Because thirteen statistical tests were performed, Bonferroni correction was applied by multiplying each *p* value by thirteen, requiring an unadjusted threshold of *p* < 0.004 to declare statistical significance. The primary predictor was the G allele frequency (percent), and latitude was defined as the absolute latitude of the population‐weighted centroid (degrees). Pearson's correlation coefficients were calculated to assess the relationships between annual OM incidence, latitude, and G allele frequency. Simple linear regressions were performed using annual OM incidence as the dependent variable and either latitude or G allele frequency as the independent variable. A multiple regression model then included both latitude and G allele frequency to determine their joint effects on incidence. Standardized regression coefficients (*β*std) were computed to compare the relative magnitude of these effects. To further quantify the unique contribution of each predictor in the multiple regression, partial correlations were calculated. The partial correlation (*r*_partial_) for a given predictor (e.g., G allele frequency) was computed from the corresponding *t*‐statistic and the residual degrees of freedom (df):

$$r_{\text{partial}}=\sqrt{\frac{t^{2}}{t^{2}+\mathrm{df}}}$$

Residual diagnostics, including Q–Q plots and residual‐versus‐fitted plots, were examined to verify the assumptions of linear regression. Scatter plots with fitted regression lines and 95% confidence intervals were generated. In addition, a mediation analysis was conducted to evaluate whether the association between latitude and OM incidence was mediated by the G allele frequency. In this analysis, latitude served as the independent variable, G allele frequency as the mediator, and OM incidence as the outcome. Bootstrapping with 1000 simulations was employed to estimate the average causal mediation effect (ACME), the average direct effect (ADE), and the proportion of the total effect mediated. All analyses were conducted using R version 4.2.2 (R Foundation for Statistical Computing, Vienna, Austria) with the dplyr, ggplot2, ggpubr, MASS, mediation, ppcor, rnaturalearth, rnaturalearthdata, and sf packages.

## 3 Results

### 3.1 Descriptive Statistics

**FIGURE 2:**
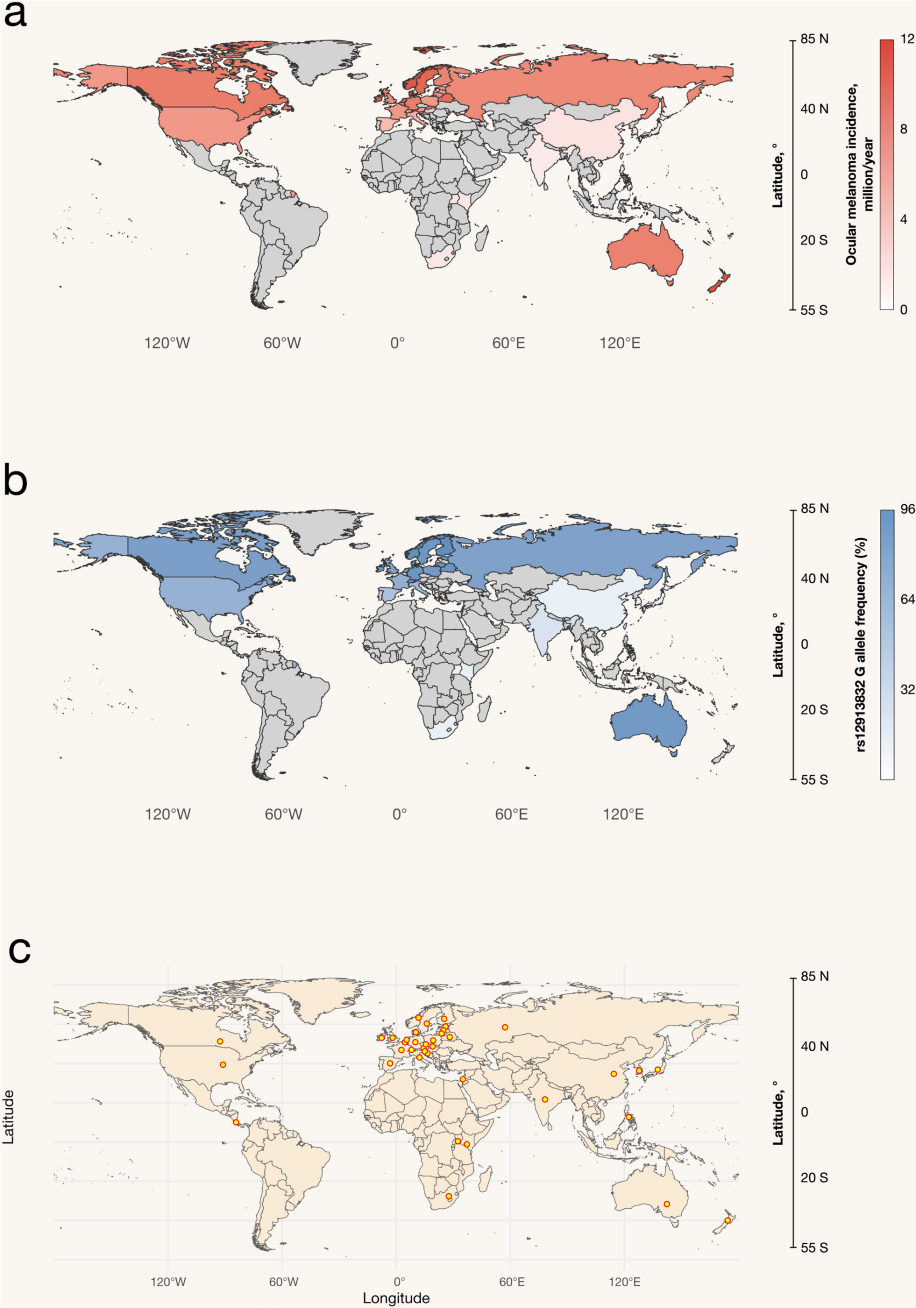
World map. (a) Ocular melanoma incidence in 36 countries; (b) Country‐level rs12913832 G allele frequency (G allele frequency). (c) Population centroids for each included country, representing the average geographic position of a country's population, accounting for internal variation in population density.

Complete data on age‐standardized incidence rates and G allele frequencies were available for 29 countries. Annual incidence rates varied markedly, ranging from 0.1 cases per million per year in India to 11.3 cases per million per year in Denmark. Likewise, the G allele frequencies spanned a wide range—from < 1% in Sub‐Saharan Africa, Japan, and South Korea to as high as 88% in Finland (Figure 2). A comprehensive summary of the collected data including population centroids is provided in Table 1.

**TABLE 1:** Summary of ocular melanoma incidence, population centroids, and G allele frequency data.

Country	Incidence (cases/million/year)	Incidence period	Population centroid (longitude, latitude)	G allele frequency (%)
Australia	7.4	2008–2012	142.3, −31.7	79^a^
Austria	6.2	2008–2012	14.3, 47.7	—
Belarus	8.7	2008–2012	28.1, 53.5	82
Belgium	7	2008–2012	4.5, 50.8	—
Canada	7.7	2008–2012	−92.7, 51.3	73^a^
China	0.6	2008–2012	114.4, 34.7	1^a^
Costa Rica	1.4	2008–2012	−84.3, 10.0	—
Croatia	5.9	2008–2012	16.4, 45.2	—
Czech Republic	6.1	2008–2012	15.5, 49.8	—
Denmark	11.3	2008–2012	10.3, 56.0	86
Estonia	6.2	2008–2012	25.9, 58.9	84^a^
Finland	7.2	2003–2007	25.2, 62.8	88
France	5.8	2008–2012	2.7, 46.9	59
Germany	7.7	2008–2012	10.0, 50.9	82
India	0.1	2008–2012	78.2, 21.7	13
Ireland	9.8	2008–2012	−7.7, 53.1	80
Israel	5.3	2008–2012	35.0, 32.0	42^a^
Italy	4.2	2008–2012	12.2, 43.1	50
Japan	0.4	2008–2012	137.5, 36.9	1^a^
Kenya	0.3	1998–2012	37.1, −1.2	1^a^
Latvia	5.4	2008–2012	24.7, 56.9	—
Lithuania	6.1	2008–2012	23.8, 55.3	82^a^
Netherlands	9.5	2008–2012	5.4, 52.1	75^a^
New Zealand	10.4	2008–2012	174.3, −40.0	—
Norway	10.1	2008–2012	11.7, 63.4	86
Philippines	0.4	2008–2012	122.3, 12.7	—
Poland	5.8	2008–2012	19.4, 51.8	78
Russia	6.9	2008–2012	57.2, 58.5	71^a^
Slovakia	8.2	2008–2012	19.1, 48.7	—
Slovenia	6.4	2008–2012	14.9, 46.2	66
South Korea	0.9	2008–2012	127.7, 36.4	1^a^
Spain	3.3	2008–2012	−3.3, 40.1	42
Sweden	9.2	2008–2012	16.0, 60.4	81^a^
Switzerland	4.1	2008–2012	8.0, 47.0	74^a^
Uganda	0.4	1988–2012	32.4, 0.4	0^a^
United Kingdom	6.6	2008–2012	−2.0, 53.1	69^a^
United States	5.9	2008–2012	−91.2, 39.4	57^a^
South Africa	0.3	1998–2012	27.6, −27.7	1^a^

Abbreviation: –, not available.

^a^ Weighted average based on the proportion of ethnically diverse population groups within the country and the G allele frequency observed in each group.

### 3.2 Correlation and Regression Analyses

**FIGURE 3:**
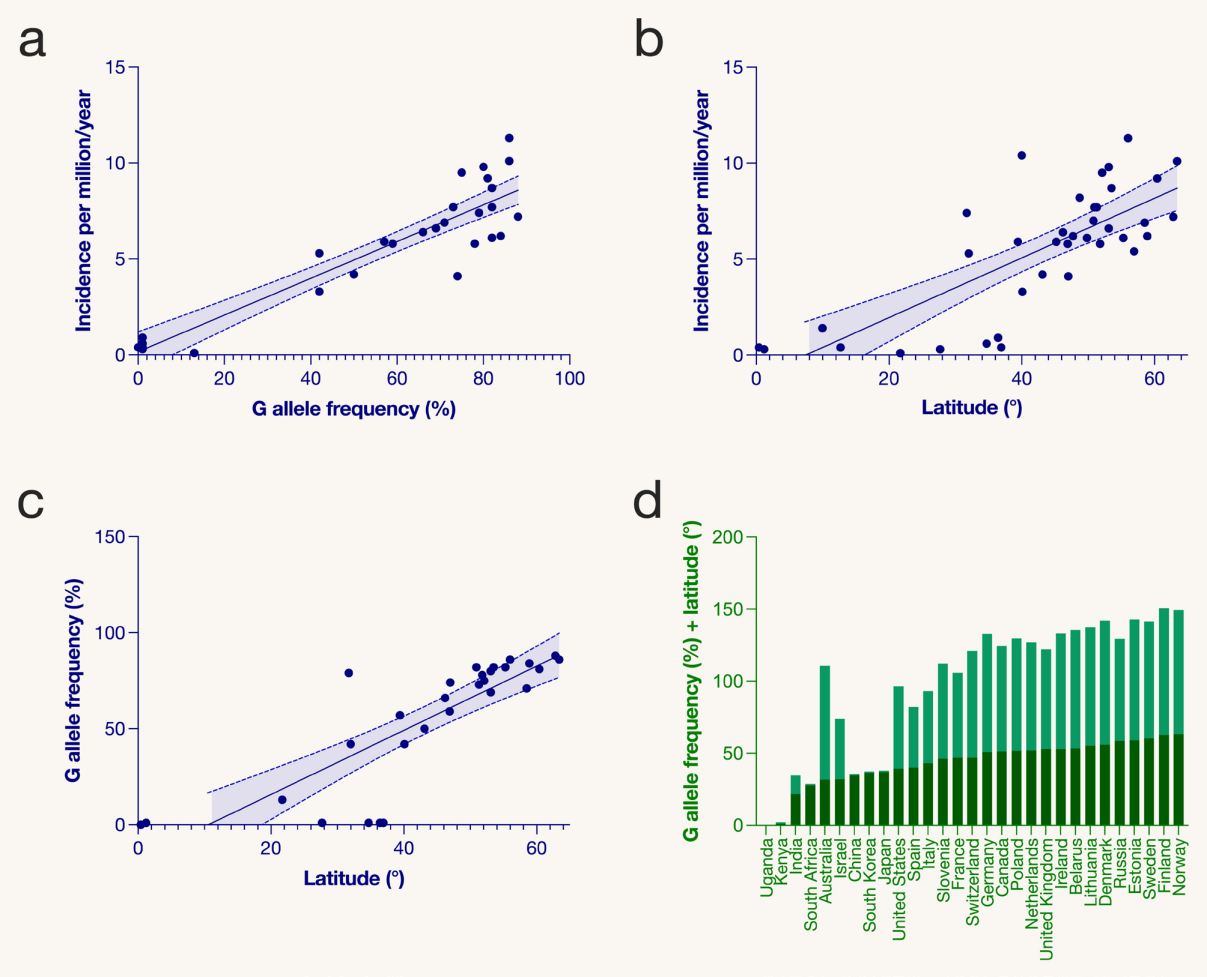
Linear regressions. (a) A 1° increase in the latitude of the population centroid was associated with an increase of 0.16 cases per million per year (SE = 0.02, *t* = 7.04, *p* < 0.001). (b) A 1 percentage point increase in rs12913832 G allele frequency was associated with an increase of 0.10 cases per million per year (SE = 0.01, *t* = 12.09, *p* < 0.001). (c) In turn, a 1° increase in latitude was associated with an increase of 1.67 percentage points in G allele frequency (SE = 0.22, *t* = 7.52, *p* < 0.001). (d) The 29 countries with complete data on both population centroids (dark green) and G allele frequency (light green) are sorted by increasing latitude, with stacked G allele frequencies and latitudes. All latitudes were converted to absolute values, such that higher values indicate a greater distance from the equator regardless of hemisphere.

Correlation analyses showed that annual OM incidence was positively associated with both latitude (*r* = 0.76, 95% CI: 0.58–0.87, *t*(34) = 7.04, *p* < 0.001) and the G allele frequency (*r* = 0.92, 95% CI: 0.83–0.96, *t*(27) = 12.09, *p* < 0.001). In simple linear regression analyses, a 1° increase in latitude was associated with an increase of 0.16 cases per million/year (SE = 0.02, *t* = 7.04, *p* < 0.001), whereas a 1 percentage point (pp) increase in G allele frequency was associated with an increase of 0.10 cases per million/year (SE = 0.01, *t* = 12.09, *p* < 0.001, Figure 3). When both predictors were included in a multiple regression model (based on 29 observations, with 9 omitted due to missing G allele frequency data), the G allele frequency remained significantly associated with incidence (*β* = 0.09, SE = 0.01, *t* = 6.40, *p* < 0.001), while latitude was no longer significant (*β* = 0.01, SE = 0.03, *t* = 0.48, *p* = 0.46). Standardized coefficients indicated that the effect of G allele frequency (*β*std = 0.90) substantially exceeded that of latitude (*β*std = 0.06). These findings suggest that although both latitude and G allele frequency are individually associated with incidence, the distribution of the rs12913832 allele is the stronger predictor of uveal melanoma incidence.

### 3.3 Partial Correlations

Next, we examined partial correlations to assess the predictive strength of each variable. G allele frequency displayed a partial correlation of approximately *r*_partial_ = 0.78 (*t* = 6.40, df = 26), indicating it accounted for about 61% of the variance in incidence not explained by latitude ($r_{\text{partial}}^{2}$ = 0.61). In contrast, latitude had a partial correlation of *r*_partial_ = 0.09 (*t* = 0.46, df = 26), corresponding to < 1% explained variance ($r_{\text{partial}}^{2}$ < 0.01). Thus, in the context of a joint model, the G allele frequency predictor uniquely explained 61 times the variance in incidence compared with latitude. Collectively, these results indicate that although both latitude and G allele frequency independently correlate with OM incidence, the distribution of the rs12913832 allele is a substantially stronger predictor once both factors are considered together.

### 3.4 Mediation Analysis

**FIGURE 4:**
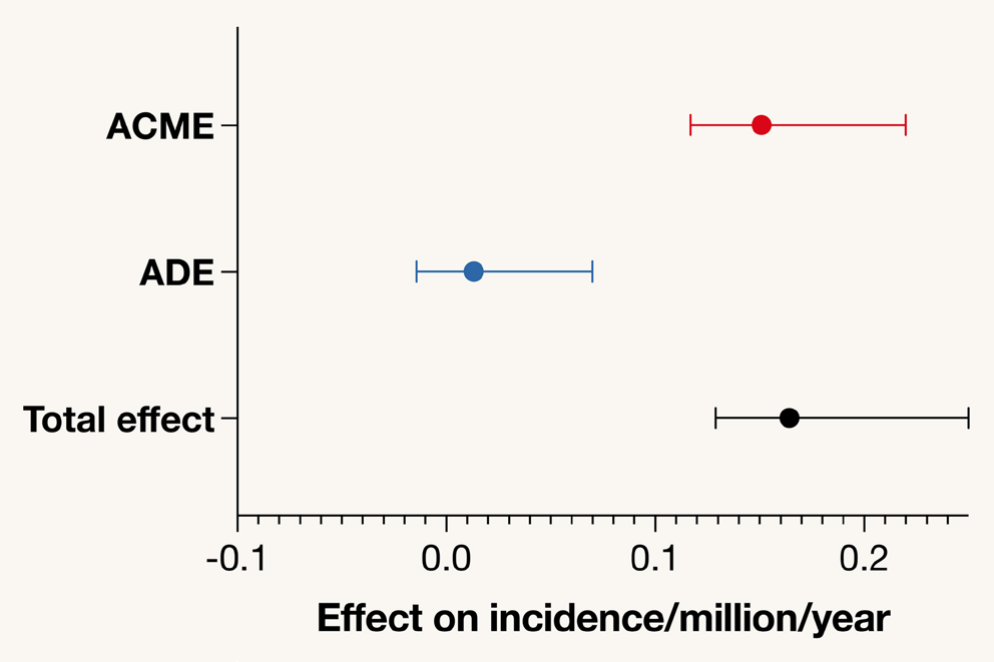
Mediation analysis. Error bars indicate the 95% confidence intervals based on 1000 simulation draws. The ACME (0.08–0.21) quantifies the average causal mediation effect, representing the portion of latitude's effect on ocular melanoma incidence (per additional degree of latitude) that is transmitted via the G allele frequency (per additional percentage point). In contrast, the ADE (−0.04 to 0.06) reflects the average direct effect—that is, the effect of latitude on incidence that is independent of G allele frequency. The total effect (0.10–0.21) is the combined impact of both the direct and mediated pathways.

The mediation analysis decomposed the total effect of latitude on ocular melanoma incidence into an indirect effect mediated by the G allele frequency and a direct effect. The ACME was estimated at 0.15 (95% CI: 0.12–0.22, *p* < 0.001), indicating that most of the effect of latitude on incidence operates through its influence on G allele frequency. In contrast, the ADE of latitude on incidence was negligible (0.01, 95% CI: −0.01–0.07, *p* = 0.31). The total effect of latitude was 0.16 (95% CI: 0.13–0.25, *p* < 0.001), with approximately 92% of this effect mediated by the G allele frequency (Figure 4). These results reinforce that the association between latitude and ocular melanoma incidence is primarily accounted for by the geographic distribution of the blue‐eye allele.

### 3.5 Sensitivity Analyses

A leave‐one‐out sensitivity analysis was conducted by iteratively excluding each country and refitting the multiple regression model. The coefficient estimates for G allele frequency remained consistently between approximately 0.089 and 0.095 (all with *p* < 0.001), while the coefficients for latitude remained non‐significant (*p* > 0.54) across all iterations. This confirms that the robust association between G allele frequency and OM incidence is not driven by any single country.

Furthermore, to assess the impact of potential errors in our G allele frequency estimates, we randomly varied each country's G allele frequency by ±25 pp (with values capped between 0 and 100%). Under these conditions, the multiple regression model yielded a significant association for G allele frequency in 9980 out of 10,000 iterations (*p* < 0.05) and for latitude in 5777 iterations (*p* < 0.05). These analyses reinforce the conclusion that the observed relationship between the G allele frequency and OM incidence is robust to plausible variations in the underlying G allele frequency data.

## 4 Discussion

Here, we demonstrate that while latitude is associated with OM incidence, this association is no longer observed after adjusting for the G allele frequency in the examined populations from 29 countries. Notably, the proportion of inhabitants with the blue‐eye allele explains the variation in OM incidence approximately 61 times better than geographic latitude, with nearly 92% of the effect of latitude on incidence mediated by the G allele frequency. In multivariate linear regression, latitude did not emerge as an independent predictor of OM incidence, and partial correlations indicated that it accounted for < 1% of the variance unexplained by G allele frequency. Thus, at the population level, the G allele frequency appears to be a much more robust predictor of the marked variation in OM incidence worldwide than geographic latitude. These population‐level results are consistent with individual‐level data: candidate‐gene and genome‐wide association studies have linked the rs12913832 G allele to increased uveal melanoma risk, with the strongest association for monosomy 3 tumors [7, 8]. The present ecological design cannot by itself demonstrate individual‐level risk, but the convergence of these two lines of work supports the interpretation that pigmentation genetics underlie the geographic variation in OM incidence.

Although ultraviolet (UV) exposure is a well‐established causal factor for cutaneous melanoma, it does not appear to be a strong direct agent for OM. This is likely because most UV radiation is absorbed by the cornea and crystalline lens, substantially limiting the amount that reaches the uveal tract [33]. The comparable incidence of uveal melanoma in Australia and Sweden—despite markedly different latitudes and UV irradiance levels but similar G allele frequencies—further supports the view that genetic factors, rather than geographic location per se, are the primary determinants of OM risk [14, 34]. Nevertheless, some UV radiation does reach the posterior segment, and carriers of the homozygous derived (GG) genotype may be more sensitive to this limited exposure [35]. Such increased sensitivity could trigger tumorigenic processes that do not display the typical UV‐related mutational signatures, potentially explaining both the absence of these signatures in posterior UM and the association of certain occupational exposures (involving high heat and UV radiation) with an increased risk of UM [19, 36]. Iris melanoma is a notable exception, in which whole‐genome sequencing has identified an ultraviolet radiation signature that is absent from choroidal and ciliary body melanoma [19].

### 4.1 Potential Mechanisms Linking rs12913832 to OM Development

The derived allele at rs12913832 downregulates OCA2 expression, leading to a reduced synthesis of eumelanin in uveal melanocytes [6]. Eumelanin not only confers darker pigmentation but also plays a critical role in absorbing harmful wavelengths of light and neutralizing reactive oxygen species [37]. Consequently, lower eumelanin levels may leave uveal melanocytes more vulnerable to oxidative stress. Although the uveal tract is less exposed to UV radiation than the skin, exposure to other wavelengths of light can still trigger oxidative stress, thereby promoting cumulative DNA damage over time. Moreover, clinical studies have observed that patients with the homozygous derived (GG) genotype more frequently exhibit UM with aggressive cytogenetic profiles and poorer prognoses [38]. In individuals with the blue‐eye genotype, the relative reduction in eumelanin compared to pheomelanin—which is more prone to generating reactive oxygen species—creates an environment conducive to oxidative damage. Pheomelanin can generate free radicals upon light exposure, and the ensuing oxidative stress may accelerate the accumulation of DNA mutations in melanocytes, thereby increasing the risk of malignant transformation [37].

Furthermore, the pigmentation pathway affects more than just iris coloration. Melanocytes in lightly pigmented eyes may demonstrate altered proliferation and dysregulated cellular homeostasis compared to those in darker eyes, potentially rendering them more susceptible to transformation [38]. The overlapping genetic networks that regulate pigmentation and melanoma susceptibility suggest that variants such as rs12913832 impact not only melanin production but also cellular responses to oxidative stress [39]. This shared genetic architecture may partly explain the higher incidence of OM observed in populations with an elevated frequency of the blue‐eye allele.

### 4.2 Evolutionary Origin

Nakagome and colleagues estimated that the region or broader selection signals related to pigmentation may have been under selection for over 42,000 years [40]. However, it is important to distinguish between a long‐standing selection signal in a genomic region and the emergence of a specific mutation. In this context, “selection signals” refer to specific patterns in the genomic data—such as reduced genetic diversity and extended haplotype homozygosity—that indicate that a genomic region has been under the influence of natural selection (i.e., “selection pressures”). Selection pressures are the environmental or cultural factors that cause differential reproductive success among individuals, leading to changes in allele frequencies over time. The derived allele of rs12913832—the one that results in reduced OCA2 expression and blue eye color—has been consistently estimated to have originated much more recently, roughly 6000–10,000 years ago [4]. The older selection signal identified by Nakagome may reflect earlier, perhaps more subtle, shifts in pigmentation‐related genetic architecture or selection on precursor variants in the region, rather than the appearance of the rs12913832 mutation itself. Therefore, while the genomic region around rs12913832 may show evidence of selection dating back over 42,000 years, the specific mutation responsible for blue eye color arose later in the evolutionary history of *Homo sapiens* . This suggests that early selection might have acted on a broader set of variants in the pigmentation pathway, with rs12913832 emerging as a key mutation in modern humans.

### 4.3 Limitations

This study uses partly the same incidence data as the previous publication by Wu and colleagues [13]. Our approach differs in three respects. First, we obtained G allele frequencies from a different repository (ALFRED). Second, we added population‐weighted centroids for each country, which reflect where the population actually resides rather than the geographic center of the landmass. Third, whereas Wu and colleagues reported correlations of OM incidence with latitude and with pigmentation‐related factors separately, we entered both latitude and G allele frequency into a single multivariable model and compared their relative contributions, complemented by mediation and sensitivity analyses. This tests which of the two is the stronger predictor of OM incidence rather than confirming that each is individually associated with it. This distinction is important given the long‐standing debate regarding the etiological significance of latitude and UV radiation in OM genesis.

Secondly, G allele frequencies were reported for specific population groups (e.g., “Ashkenazi Jews” and “Swedes”) rather than for entire countries, and these groups do not necessarily fully overlap with the national population. We attempted to mitigate this limitation by averaging the G allele frequencies when two or more groups of similar size were available within a country, or by using the values from a group representing the vast majority of a country's population (e.g., Han Chinese). Nevertheless, the G allele frequencies may not fully capture the genetic diversity within entire countries. To further address this issue, we conducted sensitivity analyses by excluding individual countries and varying the G allele frequencies by ±25 percentage points.

Thirdly, both G allele frequencies and latitudes were analyzed at the country level. Many countries span wide geographical ranges, and ideally, OM incidence and G allele frequency data would be available on a finer, regional scale (e.g., by each degree of latitude). The use of country‐level data may therefore obscure important intra‐national variations and contribute to residual confounding.

Fourthly, the ecological study design precludes direct causal inferences at the individual level (i.e., ecological fallacy). Unmeasured confounding factors—including additional genetic variants, environmental exposures, socio‐economic variables, and differences in healthcare access—could also influence OM incidence.

Fifthly, by analyzing OM as a single entity rather than focusing solely on UM, we grouped together disease entities with distinct risk factors, treatment approaches, and prognoses. For instance, conjunctival melanoma is likely more influenced by UV exposure—similar to cutaneous melanoma—than UM, potentially introducing latitude‐related effects into the G allele frequency analyses. However, since UM constitutes approximately 85% of OM cases, this impact is likely limited. Moreover, due to the organization of the underlying IARC data, it was not possible to separate UM from the other OM subtypes. Iris melanoma, which carries a distinct ultraviolet‐radiation signature, could likewise not be analyzed separately, although it represents only a small fraction of UM [19].

Finally, reliance on historical and published data introduces potential inaccuracies due to variations in data collection methods, case verification processes, and reporting biases across countries. Although sensitivity analyses support the robustness of our findings, these inherent data limitations should be considered when interpreting our results.

## 5 Conclusions

Our analyses show that, at the population level, the derived (G) allele at rs12913832 is a substantially stronger predictor of ocular melanoma incidence than geographic latitude. Nearly all the association between latitude and incidence is mediated by differences in G allele frequency, with latitude contributing minimally once genetic variation is considered. Because the study is ecological, these results identify a country‐level association and do not establish individual‐level genetic risk; the latter is, however, supported by separate candidate‐gene and genome‐wide association studies [7, 8]. Together, these findings point to pigmentation‐related genetic factors as a major correlate of the international variation in ocular melanoma incidence. Further research is warranted to clarify the biological mechanisms underlying this relationship.

## Author Contributions

**Hans Witzenhausen:** validation (equal), writing – review and editing (equal). **Hildur Helgadottir:** writing – review and editing (equal). **Veronica Höiom:** data curation (supporting), writing – review and editing (equal). **Roger Olofsson Bagge:** writing – review and editing (equal). **Gustav Stålhammar:** conceptualization (lead), data curation (lead), formal analysis (lead), investigation (lead), methodology (lead), project administration (lead), resources (lead), supervision (lead), visualization (lead), writing – original draft (lead).

## Funding

Gustav Stålhammar is supported by: The Swedish Cancer Society (20 0798 Fk); Region Stockholm (20200356); Stiftelsen Kronprinsessan Margaretas Arbetsnämnd för synskadade (2025‐092); and the Swedish Society of Medicine (SLS‐971390). The funders of the study had no role in study design, data collection, data analysis, data interpretation, or writing of the report.

## Conflicts of Interest

The authors declare no conflicts of interest.

## Supporting information


**Table S1:** Weighted average rs12913832 G allele frequencies.

**Data S1:** STROBE Statement—Checklist of items that should be included in reports of *cohort studies*.

## Data Availability

All data for this study were obtained from publicly available sources. Specifically, OM incidence data were retrieved from http://ci5.iarc.fr/Default.aspx, allele frequency data were obtained from https://alfred.med.yale.edu/ALFRED/index.jsp and population centroids were obtained from https://doi.org/10.6084/m9.figshare.9939494.1 All sources were last accessed on April 2, 2025.
