# Supplementary material for: Evolutionary Origin of Ocular Melanoma: Associations With rs12913832 G Allele Frequency and Latitude
Source: Cancer Med. 2026 Jun 11;15(6):e72038. doi: 10.1002/cam4.72038 (PMC13259966; doi:10.1002/cam4.72038)
Supplement: Supplementary file 1 — Table S1: Weighted average rs12913832 G allele frequencies. [file CAM4-15-e72038-s002.docx]

| **Supplementary Table 1.** Weighted average rs12913832 G allele frequencies. | | |
| --- | --- | --- |
| **Country** | **Ethnic breakdown, population % (G allele frequency)** | **Weighted average G allele frequency** |
| **Australia** | - English: 33.0% (86.0%) - Australian: 29.9% (86.0%) - Irish: 9.5% (80.0%) - Scottish: 8.6% (86.0%) - Chinese: 5.5% (0.0%) - Italian: 4.4% (50.0%) - German: 4.0% (82.0%) - Indian: 3.1% (13.0%) - Aboriginal: 2.9% (0.0%) - Greek: 1.7% (38.5%) - Unspecified: 4.7% (70.0%) | 79.0% |
| **Canada** | - Quebec: 24.4% (63.0%) - Atlantic provinces: 7.0% (77.0%) - Alberta: 12.4% (77.0%) - British Columbia: 14.4% (76.0%) - Ontario: 41.9% (76.0%) | 73.0% |
| **Estonia** | - Estonians: 69.1% (90.0%) - Russians: 23.7% (77.0%) - Others (Ukrainian, Other, Unspecified): 7.2% (50.0%) | 84.0% |
| **Kenya** | - Indigenous: 91.0% (0.0%) - Maasai: 2.5% (1.0%) - Non‐Kenyan: 1.0% (90.0%) | 0.9% |
| **Lithuania** | - Lithuanians: 84.6% (84.0%) - Poles: 6.5% (78.0%) - Russians: 5.0% (77.0%) - Belarusians: 1.0% (77.0%) - Others & Unspecified: 2.9% (50.0%) | 82.2% |
| **Netherlands** | - Dutch: 75.4% (82.0%) - EU (excluding Dutch): 6.4% (80.0%) - Turkish: 2.4% (39.8%) - Moroccan: 2.4% (15.0%) - Surinamese: 2.1% (0.0%) - Indonesian: 2.0% (0.0%) - Other: 9.3% (70.0%) | 74.8% |
| **Russia** | - Russians: 77.7% (77.0%) - Other groups (Tatar, Ukrainian, Bashkir, Chuvash, Chechen, Other, Unspecified): 22.3% (50.0%) | 71.0% |
| **Sweden** | - Swedes: 80.0% (96.0%) - Others (primarily Northern European, Arabic, or African descent): 20.0% (assumed; overall average = 81.0%) | 81.0% |
| **Switzerland** | - Swiss: 69.0% - German: 4.0% - Italian: 3.0% - Portuguese: 3.0% - French: 2.0% - Kosovar: 1.0% - Turkish: 1.0% - Other: 17.0% (assumed frequencies; overall average = 74.0%) | 74.0% |
| **Uganda** | - Baganda: 16.5% (0.0%) - Banyankole: 9.6% (0.0%) - Basoga: 8.8% (0.0%) - Bakiga: 7.1% (0.0%) - Iteso: 7.0% (0.0%) - Langi: 6.3% (0.0%) - Bagisu: 4.9% (0.0%) - Acholi: 4.4% (0.0%) - Lugbara: 3.3% (0.0%) - Other: 32.1% (0.0%) | 0.0% |
| **United Kingdom** | - Whites: 87.0% (82.0%) - Black/Asian/Mixed/Other: 13.0% (10.0%) | 69.0% |
| **US** | - Europeans: 62.0% (77.0%) - Hispanic/Latino: 19.0% (23.0%) - Black: 12.0% (16.0%) *(covering 93% of the population)* | 57.0% |

This table summarizes the ethnic composition, the rs12913832 G allele frequencies for each group, and the resulting weighted allele frequency for each country.
